# Domain-Adaptive Segment Anything Model for Cross-Domain Water Body Segmentation in Satellite Imagery

**DOI:** 10.3390/jimaging11120437

**Published:** 2025-12-09

**Authors:** Lihong Yang, Pengfei Liu, Guilong Zhang, Huaici Zhao, Chunyang Zhao

**Affiliations:** 1 Shenyang Institute of Automation, Chinese Academy of Sciences, Shenyang 110169, China; yanglihong@sia.cn (L.Y.); zhangguilong@sia.cn (G.Z.); hczhao@sia.cn (H.Z.); 2 University of Chinese Academy of Sciences, Beijing 100049, China; 3 School of Information Science and Engineering, Shenyang Ligong University, Shenyang 110159, China; zhaocy@sylu.edu.cn

**Keywords:** image segmentation, water body detection, domain adaptation, Segment Anything Model

## Abstract

Monitoring surface water bodies is crucial for environmental protection and resource management. Existing segmentation methods often struggle with limited generalization across different satellite domains. We propose DASAM, a domain-adaptive Segment Anything Model for cross-domain water body segmentation in satellite imagery. The core innovation of DASAM is a contrastive learning module that aligns features between source and style-augmented images, enabling robust domain generalization without requiring annotations from the target domain. Additionally, DASAM integrates a prompt-enhanced module and an encoder adapter to capture fine-grained spatial details and global context, further improving segmentation accuracy. Experiments on the China GF-2 dataset demonstrate superior performance over existing methods, while cross-domain evaluations on GLH-water and Sentinel-2 water body image datasets verify its strong generalization and robustness. These results highlight DASAM’s potential for large-scale, diverse satellite water body monitoring and accurate environmental analysis.

## 1. Introduction

Water monitoring is a critical component of environmental protection and resource management, with a growing global demand to assess water resources due to increasing scarcity and associated environmental challenges. Recent advances in remote sensing have enabled the use of satellite imagery to identify, analyze, and monitor water bodies on a large scale, including areas that are difficult to access. Accurate segmentation of water bodies from satellite imagery is essential for applications such as flood detection, water resource management, and environmental monitoring, and the development of efficient and precise segmentation techniques is vital for timely and reliable decisions [1].

Traditionally, water monitoring relied on labor-intensive ground surveys and aerial photography, which are time-consuming and limited in coverage. Classical methods using handcrafted features or specific feature extraction techniques have been employed for water body segmentation [2,3]. Recently, deep learning approaches, particularly semantic segmentation models, have demonstrated superior performance in identifying water body boundaries in satellite imagery [4,5]. However, challenges remain due to complex background clutter and the fact that water bodies are often partially obscured, limiting segmentation accuracy and hindering accurate estimation of water body extent. Moreover, water bodies captured by satellites exhibit considerable diversity in type and scale, ranging from lakes, rivers, and reservoirs to coastal seas. In addition, satellite imagery spans a wide variety of geographic regions and environmental conditions, including differences in terrain, climate, and seasonal variations; this diversity poses significant challenges for segmentation models. As a result, achieving robust cross-domain performance in satellite water body segmentation remains a critical and open problem.

In recent years, the Segment Anything Model (SAM) has gained widespread attention due to its remarkable performance in image segmentation tasks [6]. Trained on more than one billion images, SAM is capable of generating high-quality masks and demonstrates excellent generalization ability, which is particularly important in the field of satellite water body segmentation. This is because acquiring large amounts of labeled data for specific scenes can be extremely challenging. However, SAM requires prompts (such as points, boxes, and masks) to function, which makes it unsuitable for direct use in semantic segmentation tasks. Several studies have explored the application of SAM in specific domains, such as medical image segmentation and remote sensing image segmentation [7,8], showing promising results. This led us to consider whether SAM could help us tackle the complex task of segmenting satellite water bodies.

To address the challenges of cross-domain water body segmentation, we propose DASAM, a domain-adaptive Segment Anything Model for satellite imagery. The core of DASAM is a contrastive learning module that aligns features between synthetic images augmented with source–domain and style, enabling robust generalization without requiring annotations in the target domain. SAM is treated as a largely frozen backbone, while a lightweight semantic segmentation model generates coarse masks, point prompts, and bounding box prompts to provide prior knowledge. An adapter module is incorporated into SAM’s image encoder using a residual design, allowing selective parameter updates to focus on water body regions. Additionally, features from the lightweight model are fused with SAM’s encoder features to enrich semantic information for the decoder, further enhancing segmentation performance.

In summary, our contributions are as follows:

(1) We introduce a contrastive learning module built within SAM to align features between source–domain and style-augmented synthetic images, enabling robust cross-domain water body segmentation without requiring target-domain annotations.
(2) We incorporate a lightweight semantic segmentation model and insert two adapters into SAM’s image encoder, combining this with a prompt-based structure and feature map fusion to provide prior knowledge and enhance the model’s focus on water body regions.
(3) Extensive experiments on the China GF-2 dataset and cross-domain evaluations on GLH-water and Sentinel-2 datasets demonstrate that DASAM outperforms existing methods in segmentation accuracy and generalization.

## 2. Related Works

### 2.1. Segmentation Methods for Satellite Water Body Images

Before the advent of deep learning, early methods for satellite water body segmentation were largely similar to those used for natural images, typically involving pixel classification with theoretically grounded classifiers and subsequent feature extraction to derive the final segmentation [9,10,11,12,13]. For example, some studies segmented water bodies based on water pixels using spectral analysis [14,15,16]. Another study used a variety of machine learning classifiers to classify water bodies in satellite imagery [17]. However, there are significant differences between natural images and satellite images, including variations in the data acquisition process, resolution, and spectral bands. Consequently, the accuracy of these traditional methods was often insufficient, providing limited value for water resource monitoring applications.

For classification-based tasks, the widespread adoption of convolutional neural networks (CNNs) has led many studies to use various CNN-based models to classify water bodies in satellite images [18,19,20,21]. However, this approach tends to overlook the precise delineation of water body boundaries, particularly in complex or heterogeneous environments where water bodies are difficult to distinguish from other land features. Moreover, models trained on one satellite dataset often struggle to generalize to images from different sensors, resolutions, geographic regions, or environmental conditions, limiting their applicability in diverse real-world scenarios.

On the other hand, segmentation-based approaches aim to generate precise boundaries of water bodies by segmenting the entire image into regions of interest. CNNs have also shown strong performance in this regard. For example, some studies have used multiscale fusion modules within CNN architectures to improve water body segmentation in satellite images [22,23,24]. Another study utilized a deconvolutional neural network and proposed a new loss function called edge-weighted loss to improve segmentation performance [25]. Recently, some researchers have coupled Transformer networks with CNNs to improve the segmentation accuracy of satellite water body imagery [26]. Nonetheless, a common limitation in existing water body segmentation research is insufficient focus on or accuracy in delineating boundaries, as well as limited robustness and generalization across different satellite domains and environmental conditions.

### 2.2. Applications of SAM

SAM’s powerful zero-shot generalization capability has made it highly popular for image segmentation tasks since its introduction. Many fields have begun to explore their application, such as medical image segmentation [27,28], remote sensing image segmentation [29,30], and agriculture [31,32].

Researchers have explored various strategies to adapt SAM for downstream tasks. One widely used approach is adapter-based fine-tuning, where lightweight modules are inserted into SAM’s image encoder to enable task-specific adaptation without updating the full backbone. Representative examples include SAM-Adapter [33], which adapts SAM for underperformed scenes, and Medical SAM Adapter [34], which specializes SAM for medical image segmentation. Encoder-level fine-tuning methods, exemplified by Convolution Meets LoRA [35], directly update the image encoder to better capture task-specific features, which can enhance segmentation accuracy but may reduce zero-shot generalization. Prompt-based strategies, such as UVSAM [36], demonstrate that combining a small semantic segmentation model with SAM’s mask decoder via prompt generation can effectively segment complex urban–village boundaries from satellite imagery. Some studies, such as the approach by Nanni et al. [37], explore the potential of using SAM as a segmentator to enhance the performance of existing segmentation models. In this work, SAM is provided with checkpoints extracted from masks produced by mainstream segmentators, and the segmentation masks from SAM and the specialized models are then fused. This fusion of logit masks allows consistent improvement across heterogeneous datasets, including challenging tasks such as camouflage object segmentation and fine-grained object segmentation.

Compared with existing SAM-based segmentation approaches, DASAM leverages contrastive learning to explicitly align features across domains. While fusion-based approaches, such as those proposed by Nanni et al., can improve performance on heterogeneous datasets, they may still struggle with domain shifts. In contrast, DASAM’s domain-adaptive design ensures robustness across multiple satellite datasets while capturing fine-grained spatial details through its prompt enhancement and encoder adapters, enabling stronger cross-domain generalization without requiring multiple model fusions.

## 3. Materials and Methods

### 3.1. Overview

In this section, we present DASAM, a framework designed for cross-domain water body segmentation in satellite imagery by enhancing SAM with domain contrastive learning and lightweight adaptation mechanisms.

SAM is a large-scale image segmentation framework composed of three primary components: an image encoder, a prompt encoder, and a mask decoder. Its core concept is to perform segmentation efficiently via a prompt-driven mechanism. Specifically, the image encoder uses a Vision Transformer (ViT) to extract features and generate image embeddings. The prompt encoder processes both sparse prompts and dense prompts derived from masks. The mask decoder then integrates the image and prompt embeddings to generate the final segmentation mask.

SegFormer is a lightweight semantic segmentation model with an encoder–decoder architecture, designed for efficiency in resource-constrained environments [38]. The encoder, built on the Transformer architecture, extracts global contextual features through multiple layers of self-attention. The decoder, composed entirely of multilayer perceptrons, upsamples the encoded feature maps to restore spatial resolution and produce the final segmentation output.

**Figure 1:**
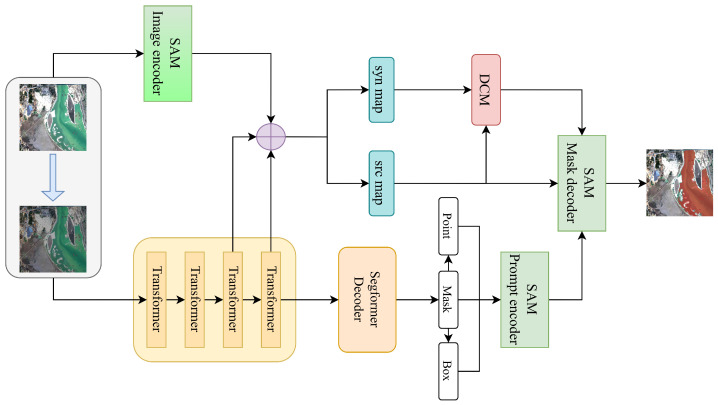
Overview of the proposed DASAM framework. Blue arrows represent the source-to-synthetic domain transformation via style augmentation, while purple circles indicate feature fusion operations.

**Figure 2:**
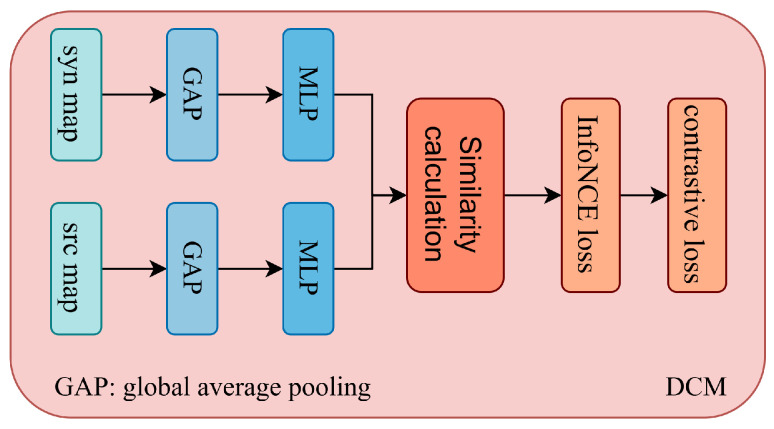
Structure of the DCM, which aligns feature distributions between source and synthetic domains through contrastive learning.

**Figure 3:**
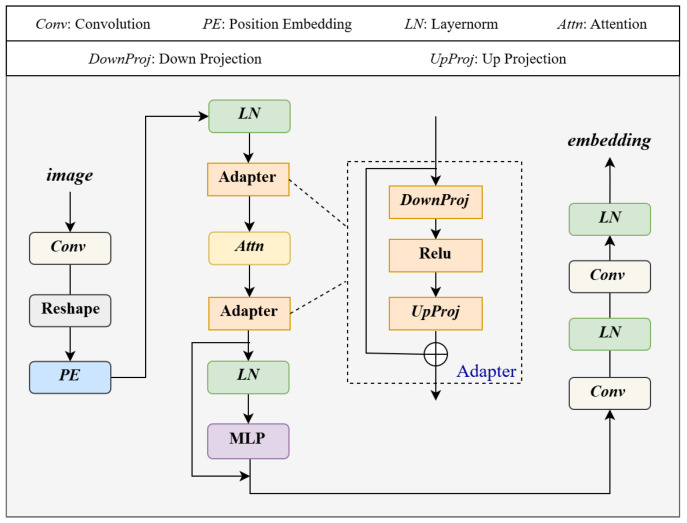
Modified architecture of SAM’s image encoder. The structure incorporates adapter modules to enable parameter-efficient fine-tuning, keeping the original pretrained weights frozen.

As illustrated in Figure 1, given a source image, we generate a synthetic style augmented image using color perturbation combined with noise injection to simulate domain variation. The color perturbation is implemented using a ColorJitter transformation with brightness, contrast, and saturation ranges set to ±0.4 and a hue variation of ±0.1. To further simulate the sensor-level domain variations, we add Gaussian noise with zero mean and a standard deviation of 0.05. Both source and synthetic images are processed simultaneously by two parallel encoders, namely the SAM image encoder and a SegFormer-based lightweight segmentation network. The SegFormer branch has two main functions. First, it provides semantic prior knowledge by producing segmentation masks from the source domain, which are used to generate prompts such as points, boxes, and masks for SAM’s prompt encoder. Second, it provides domain-aware feature maps that are fused with those extracted by SAM’s encoder to enhance feature representation and improve segmentation quality. To address cross-domain inconsistency, we introduce a Domain Contrastive Module (DCM) as shown in Figure 2. The DCM receives the fused feature pairs from the source and synthetic domains and optimizes an InfoNCE loss to encourage alignment of semantically similar representations across domains. This design enables DASAM to achieve strong generalization without requiring any annotations from the target domain. In addition, two adapter modules are inserted into the SAM image encoder, as illustrated in Figure 3. During training, the original SAM parameters remain frozen to preserve its inherent generalization capability, while only the adapter parameters are updated. The entire framework is trained on the GF-2 satellite water body dataset with a joint objective that combines segmentation loss and domain contrastive loss. The following subsections describe each component in detail.

### 3.2. Prompt

In this section, we primarily use the SegFormer model to provide prompts for SAM, including point, box, mask, and feature map prompts. Below, we provide a detailed explanation of these two types of prompts.

#### 3.2.1. Feature Map Prompt

A satellite water body image from the source domain is denoted by $I^{s}$, and its synthetic counterpart, enhanced with style, is denoted by $I^{\tilde{s}}$. Each image is processed by the SegFormer encoder, which consists of four Transformer layers with a downsampling stride of 2. The feature maps extracted from the last two Transformer layers for the source image are written as $I_{\mathrm{Seg}}^{3,s}$ and $I_{\mathrm{Seg}}^{4,s}$, and for the synthetic image as $I_{\mathrm{Seg}}^{3,\tilde{s}}$ and $I_{\mathrm{Seg}}^{4,\tilde{s}}$. The same pair of images is also fed into SAM’s image encoder to produce feature maps $I_{\mathrm{SAM}}^{s}$ and $I_{\mathrm{SAM}}^{\tilde{s}}$, which are aligned in both spatial resolution and channel dimension with the SegFormer feature maps. For each domain, the SegFormer and SAM features are integrated through a normalized additive fusion operation, formulated as

(1) $$I_{\mathrm{Seg}}^{l,d}=f_{\mathrm{Transformer}}^{l}\left(I^{d}\right),\;l=3,4,\;d\in s,\tilde{s}$$

(2) $$I_{\mathrm{SAM}}^{d}=f_{\mathrm{ViT}}\left(I^{d}\right)$$

(3) $$I_{\mathrm{fused}}^{d}=f_{\mathrm{Add}}\left(I_{\mathrm{Seg}}^{3,d},I_{\mathrm{Seg}}^{4,d},I_{\mathrm{SAM}}^{d}\right)$$

Here, $f_{\mathrm{Transformer}}^{l}$ denotes the *l*-th Transformer layer in SegFormer, $f_{\mathrm{ViT}}$ represents SAM’s image encoder, and $f_{\mathrm{Add}}$ indicates the normalized additive fusion operation. The normalized additive fusion equalizes the statistical distributions of all input feature maps via batch normalization before merging them through element-level summation. The fused feature maps $I_{\mathrm{fused}}^{s}$ and $I_{\mathrm{fused}}^{\tilde{s}}$ effectively integrate domain-specific contextual information with general visual priors from SAM. Both fused representations are subsequently utilized for downstream tasks. The source–domain fused feature $I_{\mathrm{fused}}^{s}$ is used as the input to SAM’s decoder, while the paired fused features $I_{\mathrm{fused}}^{s}$ and $I_{\mathrm{fused}}^{\tilde{s}}$ are jointly forwarded to the DCM to promote cross-domain alignment via an InfoNCE-based objective.

#### 3.2.2. Point, Box, and Mask Prompts

For the source–domain image $I^{s}$, the SegFormer model produces an initial segmentation mask, denoted as $P_{\mathrm{seg}}^{s}$. This mask provides semantic guidance for prompt generation. From $P_{\mathrm{seg}}^{s}$, we obtain its corresponding bounding box $P_{\mathrm{box}}^{s}$, which supplies spatial localization information. We randomly sample four points from the mask to form $P_{\mathrm{point}}^{s}$, including three foreground points and one background point, to represent both positive and negative spatial cues. These prompts are subsequently encoded by SAM’s prompt encoder, generating dense and sparse prompt embeddings for mask prediction. The process is expressed as

(4) $$P_{\mathrm{dense}}^{s}=f_{\mathrm{prompt}}\left(P_{\mathrm{seg}}^{s}\right)$$

(5) $$P_{\mathrm{sparse}}^{s}=f_{\mathrm{prompt}}\left(P_{\mathrm{point}}^{s},P_{\mathrm{box}}^{s}\right)=P_{\mathrm{point}}^{s}+P_{\mathrm{box}}^{s}$$

Finally, these prompts are combined with the fused feature map from the source domain to guide SAM’s decoder, producing the final segmentation result $M_{\mathrm{SAM}}^{s}$:

(6) $$M_{\mathrm{SAM}}^{s}=f_{\mathrm{decoder}}\left(I_{\mathrm{fused}}^{s},P_{\mathrm{dense}}^{s},P_{\mathrm{sparse}}^{s}\right)$$

### 3.3. Adapter

In this study, to enhance SAM’s performance in satellite water body imagery segmentation tasks, we incorporate two adapter modules into SAM’s image encoder. The adapter modules improve the model’s segmentation ability by adjusting the dimensionality of the feature representations. Specifically, we insert an adapter module before and after the Attention module in SAM’s image encoder, with identical configurations for both adapter modules.

The adapter module transforms input features *X* through a bottleneck structure involving linear downsampling, the ReLU activation function $f_{\mathrm{ReLU}}$, and upsampling, with the result integrated via residual connection to yield $X_{\mathrm{final}}$. This residual design mitigates gradient vanishing while facilitating effective feature adaptation, formulated as

(7) $$X_{\mathrm{final}}=X+\left(f_{\mathrm{ReLU}}\left(XW_{d}+b_{d}\right)W_{\mathrm{up}}+b_{\mathrm{up}}\right)$$

Here, $W_{d}$ and $b_{d}$ are the weight and bias for the downsampling operation, and $W_{\mathrm{up}}$ and $b_{\mathrm{up}}$ are the weight and bias for the upsampling operation.

### 3.4. Domain Contrastive Module

To address the distribution gap between the source and synthetic domains, we introduce a Domain Contrastive Module that performs feature-level alignment through contrastive learning. The goal of this module is to encourage the model to learn domain-invariant representations while maintaining semantic consistency across different domains.

Given a batch of source–domain images $I^{s}$ and their corresponding style-augmented synthetic images $I^{\tilde{s}}$, each synthetic image is generated by applying color perturbation and noise injection to its source counterpart. Therefore, each source image has a one-to-one correspondence with a synthetic image. After extraction and fusion of the features, we obtain the fused feature maps $I_{\mathrm{fused}}^{s}$ and $I_{\mathrm{fused}}^{\tilde{s}}$. These feature maps are passed through a global average pooling (GAP) layer to produce compact representations for contrastive learning. The pooled features are expressed as

(8) $$z^{s}=f_{\mathrm{GAP}}\left(I_{\mathrm{fused}}^{s}\right),\;z^{\tilde{s}}=f_{\mathrm{GAP}}\left(I_{\mathrm{fused}}^{\tilde{s}}\right)$$

To improve the separability of representations, we apply a projection head $f_{\mathrm{proj}}$ that maps the pooled features into a lower-dimensional contrastive embedding space. The projection head is implemented as a two-layer multilayer perceptron with ReLU activation, which transforms the 256 dimensional pooled features into 128-dimensional embeddings:

(9) $$h^{s}=f_{\mathrm{proj}}\left(z^{s}\right),\;h^{\tilde{s}}=f_{\mathrm{proj}}\left(z^{\tilde{s}}\right)$$

Assume a batch of *B* image pairs, where ${\left\{h_{i}^{s}\right\}}_{i=1}^{B}$ and ${\left\{h_{i}^{\tilde{s}}\right\}}_{i=1}^{B}$ represent the projected features of the source and synthetic domains, respectively. The pair $\left(h_{i}^{s},h_{i}^{\tilde{s}}\right)$ forms a positive sample pair, while all other combinations act as negative pairs. The similarity between two feature vectors is defined using cosine similarity with a temperature parameter τ:

(10) $$\mathrm{sim}\left(h_{i},h_{j}\right)=\frac{h_{i}\cdot h_{j}}{\tau ∥h_{i}∥∥h_{j}∥}$$

where τ is the temperature coefficient that controls the concentration level of the similarity distribution.

InfoNCE loss [39] is a contrastive learning loss that encourages positive pairs of features to be close in the feature space while pushing apart negative pairs. In our study, DCM employs a bidirectional InfoNCE loss that aligns the source and synthetic representations in both directions. For the source-to-synthetic direction, each source feature $h_{i}^{s}$ is compared with all synthetic features $h_{j}^{\tilde{s}}$, and the positive pair is defined as the corresponding synthetic feature $h_{i}^{\tilde{s}}$. The corresponding loss is expressed as

(11) $$L_{\mathrm{src}\to \mathrm{syn}}=-\frac{1}{B}\sum_{i=1}^{B}\log\frac{\exp(\mathrm{sim}\left(h_{i}^{s},h_{i}^{\tilde{s}}\right))}{\sum_{j=1}^{B}\exp\left(\mathrm{sim}\left(h_{i}^{s},h_{j}^{\tilde{s}}\right)\right)}$$

Similarly, for the synthetic-to-source direction, each synthetic feature $h_{i}^{\tilde{s}}$ treats its corresponding source feature $h_{i}^{s}$ as the positive pair, and the loss is:

(12) $$L_{\mathrm{syn}\to \mathrm{src}}=-\frac{1}{B}\sum_{i=1}^{B}\log\frac{\exp(\mathrm{sim}\left(h_{i}^{\tilde{s}},h_{i}^{s}\right))}{\sum_{j=1}^{B}\exp\left(\mathrm{sim}\left(h_{i}^{\tilde{s}},h_{j}^{s}\right)\right)}$$

The overall contrastive loss is the average of the two directional losses, written as

(13) $$L_{\mathrm{con}}=\frac{1}{2}\left(L_{\mathrm{src}\to \mathrm{syn}}+L_{\mathrm{syn}\to \mathrm{src}}\right)$$

This bidirectional contrastive formulation enforces mutual alignment between the source and synthetic domains, allowing the model to learn shared representations that are invariant to domain-specific variations. The detailed architectural configuration and hyperparameters of the DCM are summarized in Table 1.

### 3.5. Loss Function

The overall training objective combines segmentation loss and contrastive loss to achieve both accurate water body segmentation and robust domain generalization. For the segmentation task, we employ a combination of Focal loss $L_{\mathrm{focal}}$ [40] and Dice loss $L_{\mathrm{dice}}$ [41]. Focal loss addresses class imbalance by downweighting easy examples and focusing on hard negatives, while Dice loss directly optimizes the overlap between predictions and ground truth, making it particularly suitable for imbalanced segmentation tasks. The segmentation loss $L_{\mathrm{seg}}$ and total loss $L_{\mathrm{total}}$ are formulated as

(14) $$L_{\mathrm{seg}}=L_{\mathrm{focal}}+L_{\mathrm{dice}}$$

(15) $$L_{\mathrm{total}}=L_{\mathrm{seg}}+\lambda L_{\mathrm{con}}$$

where λ=0.5 is a balancing coefficient that controls the relative contribution of the two losses. The segmentation loss is computed from the SAM’s decoder output on the source–domain mask, while the contrastive loss is computed on pooled features from both domains. This joint optimization enables the model to achieve accurate water body segmentation and robust domain generalization simultaneously.

### 3.6. Dataset

The primary dataset used for model fine-tuning is the GF-2 satellite water body image dataset, which is derived from the Gaofen Image Dataset (GID) [42]. The GID dataset comprises high-resolution remote sensing images collected from over 60 cities across China using the GF-2 satellite and annotated with six land cover categories: buildings, farmland, forest, grassland, water body, and background. To adapt the dataset for binary water body segmentation, these categories were reclassified into two classes: water body and background. Images with incorrect annotations and those without any visible water body were removed. Each image has a spatial resolution of 1 m per pixel, and all images were resized to 1024 × 1024 pixels to ensure uniform input dimensions. The final dataset contains 7350 images, each paired with a corresponding binary mask. We randomly divided the dataset into training, validation, and test sets using a ratio of 6:2:2.

To further evaluate the generalization capability of the fine-tuned model, two additional satellite water body datasets, which were not involved in any training or validation, were used for external testing. These datasets vary in spatial resolution, acquisition sensors, and geographic coverage, providing a comprehensive assessment of the model’s robustness across diverse conditions.

#### 3.6.1. GLH-Water Dataset

The GLH-water dataset [43] is a globally distributed collection of very high-resolution satellite images designed for surface water segmentation. It contains 250 images acquired from diverse locations worldwide, each with a ground sampling distance of 0.3 m and a spatial size of 12,800 × 12,800 pixels, and includes manually annotated labels for water bodies. A comparison of the GLH-water dataset with the GF-2 dataset reveals several significant differences. The GLH-water dataset provides higher spatial resolution, a more diverse geographic distribution, and a wider variety of water body types and landscape contexts. These characteristics make it particularly suitable for evaluating the cross-domain generalization ability of the DASAM.

For this study, 10% of the dataset was randomly selected for testing. Each image was divided into 1024 × 1024 pixel patches, resulting in 144 tiles per original image, to match the input format of the model while preserving high-resolution details.

#### 3.6.2. Sentinel-2 Satellite Water Body Image Dataset

The Sentinel-2 Satellite Water Body Image Dataset [44] comprises 2841 Sentinel-2 satellite images, each of which is accompanied by a binary mask that distinguishes between water and non-water regions. These masks were generated using the Normalized Water Difference Index, with an elevated threshold applied to improve the detection of water bodies. Compared with the GF-2 dataset, the main differences lie in sensor type, spatial resolution, and mask generation method. Sentinel-2 images have a coarser spatial resolution, and their water masks are derived from spectral indices rather than manual annotation. Additionally, the dataset captures a variety of environmental and atmospheric conditions, resulting in diverse appearances of water bodies. These differences make the dataset particularly suitable for evaluating the zero-shot generalization of the fine-tuned SAM, as it tests the model’s robustness to variations in sensor characteristics, resolution, and water body representation.

Similarly, 10% of this dataset was held out for zero-shot testing to evaluate the model’s performance on unseen spectral and spatial characteristics. Prior to this, all images were subjected to preprocessing and resized to 1024 × 1024 pixels in order to ensure compatibility with the model input configuration.

Although the Sentinel-2 and GLH-water images are resized to standardized 1024 × 1024 patches before being fed into the model, this preprocessing step does not adversely affect the segmentation quality. For Sentinel-2 imagery, the original spatial resolution is substantially coarser than the network’s receptive field, meaning that resizing primarily serves to standardize input dimensions rather than alter the semantic structure of water bodies. For the GLH-water dataset, each 12,800 × 12,800 scene is partitioned into 1024 × 1024 patches. This strategy is widely adopted in high-resolution remote sensing segmentation, as it preserves the local spatial structure of water bodies while avoiding the distortions that would occur if the entire scene were aggressively downsampled. The resizing is applied uniformly within each tile and does not modify the relative geometry of water–land boundaries.

The incorporation of these two external datasets enables a systematic evaluation of the generalization performance of the DASAM model. The disparities in spatial resolution, geographic distribution, and imaging conditions across these datasets allow for a comprehensive analysis of the model’s robustness and adaptability in diverse real-world satellite water body segmentation scenarios.

### 3.7. Comparative Methods and Evaluation Metrics

To evaluate the effectiveness of the proposed method in satellite water body segmentation, we compared our model against several representative segmentation approaches, including DeepLabv3+ [45], Segformer, PVTv2 [46], SAM-Adapter, and UVSAM.

We adopted four widely used evaluation metrics to assess segmentation performance: F1 score (F1), recall, precision, and Intersection over Union (IoU). A true positive is a water body pixel in the ground truth that is correctly identified by the model. A false positive is a non-water pixel that the model mistakenly labels as water. These values were used to compute precision, recall, and F1. IoU was calculated as the ratio between the intersection and the union of the predicted water body mask and the corresponding ground truth mask. The specific formulas for each metric are as follows:

(16) $$\mathrm{Precision}=\frac{T_{P}}{T_{P}+F_{P}}$$

(17) $$\mathrm{Recall}=\frac{T_{P}}{T_{P}+F_{N}}$$

(18) $$F1=\frac{2\times \mathrm{Precision}\times \mathrm{Recall}}{\mathrm{Precision}+\mathrm{Recall}}$$

(19) $$\mathrm{IoU}=\frac{T_{P}}{T_{P}+F_{P}+F_{N}}$$

where $T_{P}$, $F_{P}$, and $F_{N}$ are the number of correct water body detection pixels, incorrect water body detection pixels, and undetected water body pixels, respectively.

### 3.8. Training Details

All experiments were implemented using the PyTorch 2.0.1 (Meta AI, Menlo Park, CA, USA) deep learning framework on a high-performance computing server equipped with dual Intel Xeon Platinum 8350C CPUs and an NVIDIA A100-SXM4-80GB GPU. The model was trained for 100 epochs with a batch size of 16. We employed the AdamW optimizer along with a cosine annealing learning rate scheduler to gradually reduce the learning rate during training. The initial learning rate was set to $2\times 10^{-4}$, with a weight decay of $1\times 10^{-4}$. A linear warm-up phase was applied for the first 5000 steps to stabilize the optimization process. Mixed precision training (FP16) was utilized to accelerate computation and reduce memory consumption; the peak GPU memory usage was approximately 52.7 GB. Based on our experimental results, training one epoch took approximately 577 s, while the inference time for a single image is about 0.34 s.

To evaluate the influence of the temperature parameter on the effectiveness of the DCM, we conducted a hyperparameter analysis on the temperature in the InfoNCE loss. Specifically, we evaluated temperature values of 0.05, 0.10, 0.15, and 2.0, and the results are reported in Table 2. A smaller temperature encouraged the model to emphasize harder negative samples, which strengthened discrimination but also introduced instability. This behavior was reflected in our experimental results. Although τ=0.05 produced a slightly higher IoU than τ=0.10, its F1 score decreased more noticeably. As the temperature increased beyond 0.10, both IoU and F1 showed a consistent downward trend. Considering the overall balance between accuracy and stability, we adopted τ=0.10 as the default setting for the DCM.

## 4. Results

### 4.1. Comparison with Other Methods

To provide a more reliable assessment of model performance, we further estimated the statistical variability of DASAM by performing 1000 bootstrap resamplings on the test set. The results show that the IoU of DASAM exhibits a narrow 95% confidence band ranging from 0.829 to 0.836, while the F1 has a corresponding band from 0.907 to 0.913. These intervals indicate that the performance of DASAM remains stable across different resampled subsets of the data. Combined with the quantitative results in Table 3, these findings confirm that DASAM maintains the strongest overall performance among all compared models. Specifically, DASAM achieves the highest IoU of 0.832, slightly surpassing PVTv2 by 0.3% and SegFormer by 0.9%. The F1 reaches 0.909, representing the best result among all competitors. In terms of precision, DASAM attains 0.924, ranking second only to SegFormer, while maintaining a competitive recall of 0.894. Although its recall is marginally lower than PVTv2, DASAM provides a more favorable balance between precision and recall, indicating stronger robustness in reducing false positives while preserving segmentation completeness. Table 4 further presents the validation results, where DASAM consistently achieves the highest IoU of 0.829 and F1 of 0.905 among all models. Similar to the test set, DASAM sustains strong performance across all key metrics, highlighting the effectiveness of our contrastive domain alignment strategy in enhancing model generalization.

### 4.2. Qualitative Results

**Figure 4:**
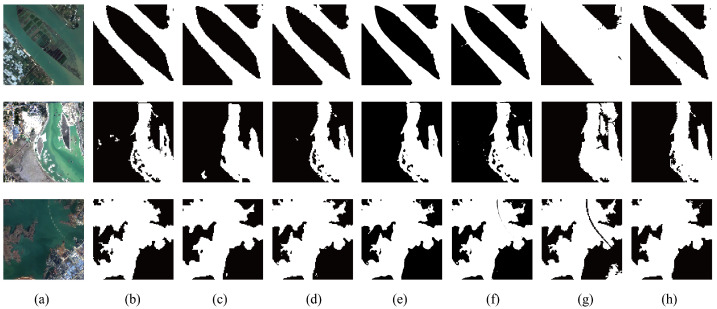
The visualization results: (**a**) Raw image, (**b**) Ground truth, (**c**) DeepLabv3+, (**d**) Segformer, (**e**) PVTv2, (**f**) SAM-Adapter, (**g**) UVSAM, (**h**) DASAM.

In this section, to further validate the effectiveness of the proposed method, we conducted a qualitative comparison of segmentation results between DASAM and several baseline models. Figure 4 presents three representative satellite water body images along with the corresponding segmentation outputs. As illustrated, DASAM consistently achieves superior visual performance in most cases.

In the first row of Figure 4, the narrow water channels exhibit high spectral similarity to surrounding buildings, making accurate segmentation particularly challenging. However, DASAM precisely delineates the water boundaries while effectively suppressing false positives and false negatives. In contrast, UVSAM shows significant segmentation errors in these areas. In the second row, the water body is surrounded by artificial structures and bare soil, the latter introducing additional interference due to its high spectral reflectance. Similarly, the third row features a water body with complex, irregular boundaries. Most baseline methods fail to capture these fine-grained details; for instance, SAM-Adapter and UVSAM produce blurred boundaries, while Segformer and PVTv2 generate excessive false positives. In comparison, DASAM accurately captures boundary structures and produces smooth, coherent segmentation maps, demonstrating superior boundary detection capabilities and overall robustness.

### 4.3. Ablation Study

To validate the effectiveness of each module and design choice in the DASAM framework, we conducted a series of ablation experiments to evaluate the performance of different variants on the GF-2 satellite water body dataset.

Table 5 presents the performance of different DASAM variants in terms of IoU, F1, recall, and precision. The results clearly demonstrate that removing any single module leads to a decline in segmentation performance, indicating that each component contributes critically to the overall architecture.

Specifically, removing point prompts reduces IoU by 2.8% and F1 by 7.4%. This highlights their importance in providing precise positional cues. The utility of point prompts is especially evident in complex scenes, where they help mitigate undersegmentation and preserve fine water body structures. Box prompts contribute substantially to model performance, yielding a 22.7% improvement in IoU by providing global spatial constraints that guide the mask decoder toward target regions and effectively suppress false positives. Although mask prompts have a relatively smaller impact, their removal still reduces IoU by 1.0%. This suggests that dense mask embeddings help refine boundary delineation and complement the global contextual understanding.

The DCM proves essential for robust feature learning, as its removal results in a 7.1% decrease in IoU and a 6.8% reduction in F1 score. This significant performance drop underscores DCM’s crucial role in learning domain-invariant representations through contrastive alignment between source and synthetic domains. The module enhances model generalization by reducing the distribution gap across different satellite imaging conditions, thereby improving segmentation consistency in varied scenarios.

In addition, the feature fusion module enhances the smoothness and continuity of segmentation results. Its removal causes a 2.5% decline in IoU and a 0.4% decrease in F1, indicating its role in semantic aggregation and cross-scale feature alignment. The adapter module yields the most significant improvements, increasing IoU by 6.2% and the F1 by 5.7%. This is because it effectively integrates fine-grained spatial details with global contextual information.

### 4.4. Generalization Evaluation

To further validate the robustness and generalization ability of the proposed DASAM model, two additional datasets with distinct characteristics were employed for evaluation.

As shown in Table 6, the proposed DASAM achieves the best overall performance on the GLH-water dataset, demonstrating its strong cross-domain generalization capability. Specifically, DASAM achieves the highest IoU of 0.484 and F1 of 0.652, outperforming all baseline models by a significant margin. Although its recall of 0.509 is slightly lower than that of UVSAM, its precision of 0.908 is substantially higher than all baselines. This indicates that DASAM effectively suppresses false positives and maintains stable segmentation quality under diverse imaging conditions.

**Figure 5:**
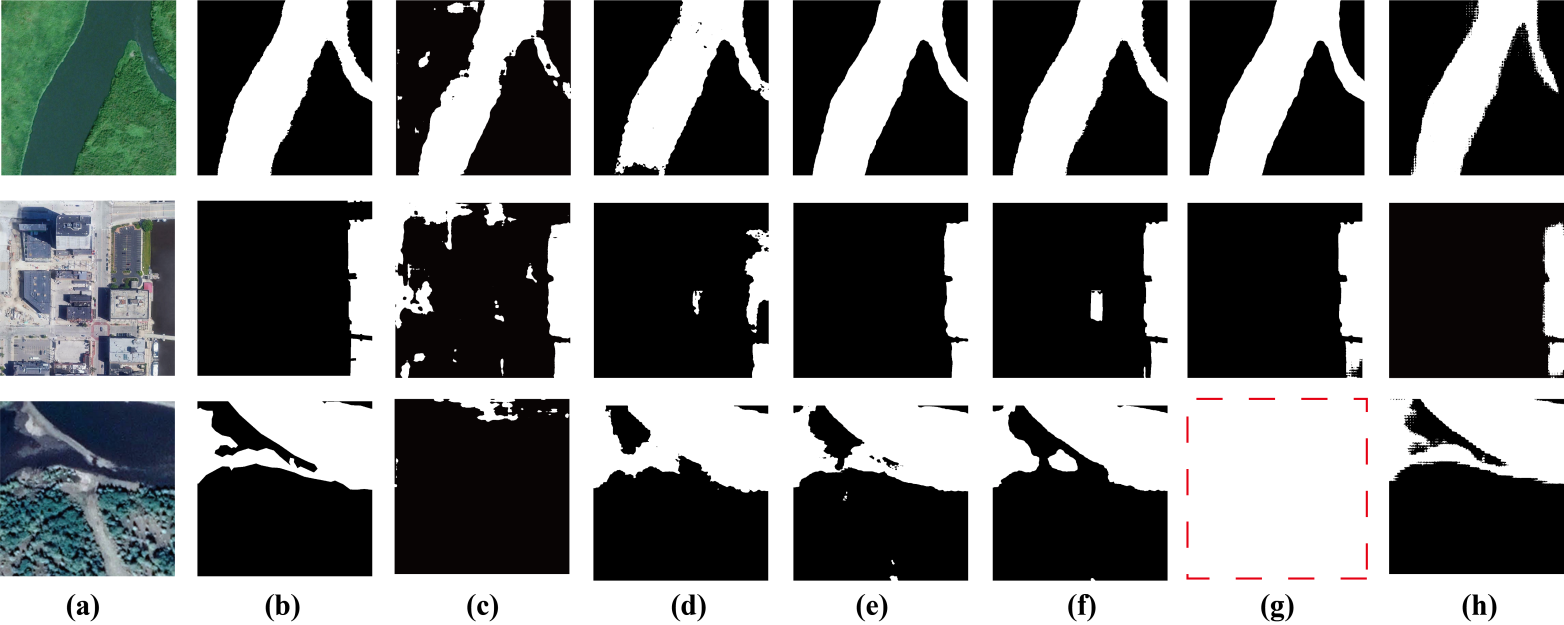
The GLH-water Dataset visualization results: (**a**) Raw image, (**b**) Ground truth, (**c**) DeepLabv3+, (**d**) Segformer, (**e**) PVTv2, (**f**) SAM-Adapter, (**g**) UVSAM, (**h**) DASAM.

The visual results in Figure 5 further substantiate the quantitative findings. The GLH-water dataset encompasses diverse geographic and environmental contexts, including natural rivers, agricultural zones, and urban–river intersections, which differ substantially from the GF-2 training data. In natural river scenes, most models generate relatively consistent segmentation, while DeepLabv3+ and Segformer produce fragmented and discontinuous predictions. In the second example, where the river adjacent to agricultural land appears darker and spectrally distinct from the training water samples, DASAM maintains precise and continuous water boundaries. In contrast, SAM-Adapter exhibits evident over-segmentation, misclassifying large non-water regions as water, while Segformer suffers from pronounced under-segmentation. These visual observations are consistent with the quantitative metrics; UVSAM yields higher recall but lower precision, while DASAM demonstrates the opposite pattern. In the third example, depicting complex urban–river scenes where shadows and dark construction materials share spectral similarities with water, segmentation becomes more challenging. Nevertheless, DASAM still produces coherent and accurate delineations, while UVSAM shows extensive false positives, and Segformer and PVTv2 misclassify several shadowed regions.

Following the evaluation on the GLH-water dataset, the model performance was further assessed on the Sentinel-2 dataset to examine generalization across domains with greater spectral and spatial similarity to the GF-2 training data. As shown in Table 7, all models achieve improved segmentation metrics compared with their performance on the GLH-water dataset, indicating that Sentinel-2 shares more consistent water body characteristics with the training domain. Nevertheless, DASAM still achieves the best overall performance, achieving an IoU of 0.537 and an F1 of 0.699, surpassing PVTv2, the second-best model, by 2.5% in IoU.

**Figure 6:**
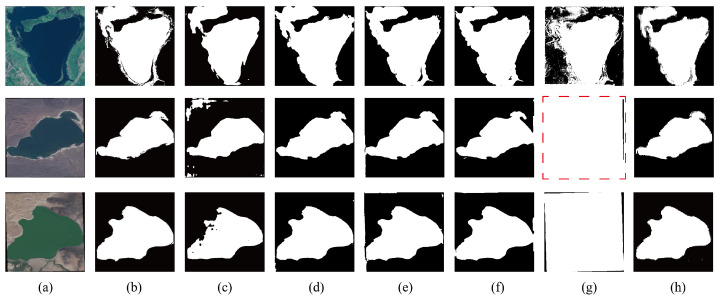
The Sentinel-2 Water Body Image Dataset visualization results: (**a**) Raw image, (**b**) Ground truth, (**c**) DeepLabv3+, (**d**) Segformer, (**e**) PVTv2, (**f**) SAM-Adapter, (**g**) UVSAM, (**h**) DASAM.

The visual comparisons in Figure 6 further confirm these results. In the first example, where both the water and background features closely resemble those in the training data, all models deliver visually plausible predictions, while DASAM exhibits more precise boundary delineation. The second and third examples depict mixed land–lake scenes, with deeper and lighter water tones, respectively, presenting increased intra-class variation. DASAM maintains robust segmentation quality under these conditions, accurately distinguishing water regions with minimal false positives. By contrast, DeepLabv3+ and UVSAM again suffer from severe over-segmentation, labeling most of the image as water, which corresponds to their high recall but low precision. In addition, both PVTv2 and SAM-Adapter produce scattered false positives in non-water areas, particularly in darker or shadowed regions, suggesting their limited adaptability to subtle spectral variations.

Overall, the cross-dataset evaluation on GLH-water and Sentinel-2 demonstrates that DASAM achieves consistently strong generalization across diverse spatial patterns and spectral domains. Even in challenging conditions, such as urban–river intersections, cultivated areas with complex textures, or lakes exhibiting significant spectral shifts, DASAM maintains stable segmentation accuracy and preserves boundary integrity. These results highlight the model’s capacity to effectively transfer its learned representation to unseen remote sensing scenarios, validating its robustness and adaptability for real-world water body extraction tasks.

## 5. Discussion

Our experimental results demonstrate the effectiveness of DASAM in enhancing SAM’s capability for satellite water body extraction across diverse domains. The SegFormer provides comprehensive prompts that effectively guide SAM to focus on water regions. This prompt-enhanced mechanism enriches the semantic priors of the framework and improves its adaptability to complex spatial structures.

The adapter module enables efficient domain adaptation while keeping SAM’s pretrained encoder intact, reducing computational cost and mitigating overfitting on limited remote sensing data. The feature fusion between SegFormer and SAM encoders enables DASAM to combine global context with fine-grained details, which is crucial for delineating irregular or fragmented water boundaries. Our analysis suggests that this design helps maintain generalizable structural priors while selectively adjusting high-level semantic cues relevant to water bodies, striking an effective balance between adaptability and stability.

Another critical component of DASAM is the DCM, which explicitly aligns feature distributions between source and synthetic domains. This mechanism is particularly beneficial for cross-domain robustness. Satellite images vary dramatically across sensors, seasons, and acquisition conditions. By enforcing domain-invariant representation learning, the DCM mitigates such discrepancies and leads to consistently improved performance on GLH and Sentinel-2 datasets. This result highlights the capability of contrastive alignment to address spectral and appearance differences that traditional supervised segmentation cannot easily overcome.

Despite these strengths, several limitations remain. Although the SegFormer prompting strategy enhances semantic focus, its performance may degrade when the initial coarse segmentation from SegFormer is inaccurate, particularly in scenes with extreme turbidity or thin river networks. In addition, the current contrastive learning objective operates only on synthetic domain pairs; extending it to real multi-domain scenarios could further improve robustness. Future work may explore more efficient prompting mechanisms and broader domain alignment strategies that incorporate additional sensing modalities such as SAR imagery. Moreover, integrating uncertainty estimation into the pipeline may further enhance reliability for practical hydrological monitoring applications.

## 6. Conclusions

This study presents DASAM, a domain-adaptive SAM framework tailored for robust satellite water body segmentation. By integrating a SegFormer-based prompt generator, encoder adapters, multi-scale feature fusion, and a novel domain contrastive module, DASAM effectively learns domain-invariant representations while capturing both global context and local details. The contrastive learning strategy aligns feature distributions across domains, enabling the model to maintain consistent performance under varying satellite imaging conditions.

On the GF-2 dataset, the model achieves an IoU of 0.832 and an F1 score of 0.909, demonstrating clear advantages over both classical baselines. The ablation study further verifies the contribution of each component, with noticeable performance declines observed whenever any module is removed. In particular, the DCM plays a central role in the framework; removing it results in a substantial drop in IoU from 0.832 to 0.761, underscoring its critical importance in enabling domain-adaptive representation learning. These consistent numerical reductions collectively indicate that DASAM benefits from the synergistic interplay among structured prompting, cross-encoder feature fusion, and contrastive domain alignment.

DASAM also exhibits strong generalization capability when transferred to unseen satellite imagery. On the GLH-water dataset, the model achieves an IoU of 0.484 and an F1 of 0.652, outperforming all compared methods by a clear margin. Similar improvements are observed on the Sentinel-2 dataset, where DASAM attains an IoU of 0.537 and an F1 of 0.699, again surpassing existing baselines. These cross-domain results highlight that one of the key contributions of this work lies in the ability of DASAM to maintain stable segmentation performance despite substantial variations in sensor characteristics, spectral properties, and scene distributions, demonstrating its robustness and practical value for real-world hydrological monitoring.

Overall, DASAM offers a practical and extensible framework for adapting large foundation models to remote sensing applications, effectively addressing the domain shift challenge in satellite imagery analysis, and future work will explore extensions to multi-temporal and multimodal settings to further enhance robustness under diverse Earth observation environments.

## Figures and Tables

**Table 1:** Architectural configuration of the proposed DCM.

Component	Setting
Input feature map	256 channels
GAP	256-d vector
MLP	256 → 128
Similarity function	Cosine similarity
Temperature τ	0.1
Loss function	Bidirectional InfoNCE

**Table 2:** Performance under different temperature values τ in the InfoNCE objective.

Temperature τ	IoU	F1
0.05	0.834	0.903
0.10	0.832	0.909
0.15	0.830	0.906
0.20	0.823	0.897

**Table 3:** Performance comparison between DASAM and other models on the test set. Bold numbers indicate the best results, while underlined numbers indicate the second-best results.

Methods	IoU	F1	Recall	Precision
DeepLabv3+	0.798	0.884	0.853	0.918
SegFormer	0.823	0.904	0.884	**0.925**
PVTv2	0.829	0.907	**0.905**	0.909
SAM-Adapter	0.806	0.892	0.895	0.889
UVSAM	0.764	0.854	0.815	0.897
DASAM	**0.832**	**0.909**	0.894	0.924

**Table 4:** Performance comparison between DASAM and other models on the validation set. Bold numbers indicate the best results, while underlined numbers indicate the second-best results.

Methods	IoU	F1	Recall	Precision
DeepLabv3+	0.795	0.882	0.844	0.924
Segformer	0.821	0.901	0.875	**0.930**
PVTv2	0.825	0.904	**0.896**	0.912
SAM-Adapter	0.813	0.897	0.894	0.900
UVSAM	0.768	0.854	0.813	0.900
DASAM	**0.829**	**0.905**	0.883	0.929

**Table 5:** Ablation study of DASAM variants on the GF-2 satellite water body dataset. Bold numbers indicate the best results.

Variants	IoU	F1	Recall	Precision
w/o Point	0.804	0.835	0.843	0.827
w/o Box	0.605	0.773	0.676	0.902
w/o Mask	0.822	0.903	0.884	0.923
w/o Fusion	0.807	0.905	**0.921**	0.890
w/o Adapter	0.770	0.852	0.859	0.845
w/o DCM	0.761	0.841	0.813	0.872
DASAM	**0.832**	**0.909**	0.894	**0.924**

**Table 6:** Performance comparison between DASAM and other models on the GLH-water Dataset. Bold numbers indicate the best results.

Methods	IoU	F1	Recall	Precision
DeepLabv3+	0.232	0.337	0.416	0.344
Segformer	0.432	0.603	0.607	0.599
PVTv2	0.383	0.554	0.484	0.648
SAM-Adapter	0.377	0.574	0.521	0.638
UVSAM	0.281	0.491	**0.880**	0.340
DASAM	**0.484**	**0.652**	0.509	**0.908**

**Table 7:** Performance comparison between DASAM and other models on the Sentinel-2 satellite water body image dataset. Bold numbers indicate the best results.

Methods	IoU	F1	Recall	Precision
DeepLabv3+	0.460	0.631	0.721	0.560
Segformer	0.503	0.669	0.675	0.664
PVTv2	0.512	0.677	**0.768**	0.606
SAM-Adapter	0.487	0.615	0.663	0.574
UVSAM	0.271	0.427	0.747	0.299
DASAM	**0.537**	**0.699**	0.665	**0.737**

## Data Availability

The code presented in this study are available on request from the corresponding author due to copyright restrictions imposed by the funding agency and The GF-2 satellite water body image dataset can be accessed at https://pan.baidu.com/s/1Mrm5JXA0INCG1dXKSOSStA?pwd=j8hi (accessed on 5 December 2025).

## Abbreviations

The following abbreviations are used in this manuscript:

SAM	Segment Anything Model
VIT	Vision Transformer
DCM	Domain Contrastive Module
GAP	Global Average Pooling
CNNs	Convolutional neural networks
